# Production and Role of Free Radicals and Reactive Oxygen Species After Facial Nerve Injury

**DOI:** 10.3390/antiox14040436

**Published:** 2025-04-04

**Authors:** Jeongmin Lee, Joon Hyung Yeo, Sung Soo Kim, Jae Min Lee, Seung Geun Yeo

**Affiliations:** 1 Department of Medicine, College of Medicine, Kyung Hee University Medical Center, Seoul 02447, Republic of Korea; 2 Public Health Center, Danyang-gun 27010, Republic of Korea; joonhyungyeo@gmail.com; 3 Department of Biochemistry and Molecular Biology, College of Medicine, Kyung Hee University, Seoul 02447, Republic of Korea; sgskim@khu.ac.kr; 4 Department of Otorhinolaryngology Head & Neck Surgery, College of Medicine, Kyung Hee University Medical Center, Seoul 02447, Republic of Korea; 5 Department of Precision Medicine, Graduate School, Kyung Hee University, Seoul 02447, Republic of Korea; 6 Department of Convergence Medicine, College of Medicine, Kyung Hee University, Seoul 02447, Republic of Korea

**Keywords:** facial nerve injury, reactive oxygen species, free radicals, oxidative stress, nerve regeneration, antioxidants

## Abstract

Facial nerve injury (FNI) induces complex molecular and cellular responses, with reactive oxygen species (ROS) and free radicals (FRs) playing pivotal roles in nerve degeneration and regeneration. However, to date, no systematic review has specifically investigated the involvement of ROS and FRs in FNI. To address this unmet need, we reviewed the literature on the subject, comprehensively searching SCOPUS, PubMed, Cochrane Library, EMBASE, and Google Scholar to identify studies that assessed the roles of FRs and ROS in FNI and summarize their findings. A total of 15 studies that satisfied search criteria were identified. Key findings showed that excessive ROS and FR lead to mitochondrial dysfunction, lipid peroxidation, and ferroptosis, exacerbating nerve degeneration after facial nerve injury. These effects are modulated by antioxidants, including alpha-lipoic acid, edaravone, N(ω)-nitro-L-arginine methyl ester (L-NAME), glutathione peroxidase 4, glutathione, methylprednisolone sodium succinate, Si-based agents, superoxide dismutase, and tirilazad mesylate. The insights gained from this review suggest that levels of FRs and ROS are strongly associated with the pathophysiology of facial nerve injury and underscore the therapeutic potential of targeting ROS and FR pathways in facial nerve injuries.

## 1. Introduction

### 1.1. Facial Nerve

The facial nerve emerges between the pons and the medulla oblongata and travels alongside the vestibulocochlear nerve through the internal acoustic meatus. It then separates at the base of the internal acoustic meatus, with only the facial nerve entering the facial canal. Beyond the geniculate ganglion, the facial nerve bends downward, extending branches to the stapedius muscle and chorda tympani nerve, and exiting the temporal bone through the stylomastoid foramen. It then becomes distributed among facial expression muscles in the face and neck through its temporal, zygomatic, buccal, marginal mandibular, and cervical branches. The facial nerve can be divided into upper and lower parts relative to the motor nucleus. The upper part of the motor nucleus receives both crossed and uncrossed fibers from the pons, innervating both sides of the face, whereas nerve fibers in the lower part of the motor nucleus are only innervated by the contralateral cerebral cortex. Thus, because the upper face receives bilateral innervation, an upper motor nucleus lesion on one side causes paralysis that mainly appears in the lower facial muscles around the mouth, without affecting muscles controlling the upper face (e.g., forehead) or the orbicularis oculi. Taste, saliva secretion, and tear secretion also remain unaffected. Conversely, in the case of a lower motor nucleus lesion on one side, both upper and lower facial muscles on the affected side are completely paralyzed [1,2,3].

Facial nerve paralysis can result from a variety of conditions, including infections; trauma; tumors; and congenital, idiopathic, metabolic, and systemic diseases. Whereas most conditions cause acute paralysis, tumors or cholesteatomas lead to progressive paralysis [4,5,6,7,8]. Although facial nerve paralysis is not a life-threatening condition, it can be devastating for an individual’s emotional and social life, making treatment outcomes and prognosis extremely important. The prognosis for patients with facial nerve paralysis is determined by the cause of the condition, the extent of the damage, and the treatment options available to the patient. While there is considerable debate regarding the indications, timing, and approaches for facial nerve decompression and surgical treatment method, clinicians dealing with facial nerve paralysis can improve patient outcomes by thoroughly understanding the surgical indications, treatment methods, and rehabilitation therapies applicable to patients with facial paralysis [9,10,11,12,13].

### 1.2. Reactive Oxygen Species

Research on reactive oxygen species (ROS) has advanced rapidly since the initial discovery of superoxide dismutase (SOD), despite the inherent challenge of identifying individual ROS owing to their high reactivity and rapid interactions with surrounding substances. In the decades since, extensive research has been conducted on ROS across various fields, including physical chemistry, biochemistry, radiology, medicine, and botany, as well as cellular immunology, genetics, and the development and inhibition of cancer [14,15,16].

The known types of ROS include superoxide radical [or anion] (O_2_^−^), perhydroxyl radical (HO_2_), hydrogen peroxide (H_2_O_2_), hydroxyl radical (HO·), singlet oxygen (^1^O_2_), hypochlorite (OCl^−^), and ozone (O_3_). Various pathways generate ROS in the body; it is estimated that, even with normal metabolic processes, 2–3% of the oxygen we breathe is converted into superoxide radicals (O_2_^−^). Thus, although 97–98% of the oxygen we inhale has a beneficial effect on cellular metabolism, the remaining 2–3% can be detrimental. In addition to normal metabolic processes, harmful environmental factors such as ultraviolet rays, pesticides, cigarette smoke, and air pollution can also generate ROS. ROS produced in our bodies as a result of activity and respiration react with the unsaturated fatty acids of cell membranes to form new radicals known as peroxy radicals [17,18,19].

The human body has an efficiently structured antioxidant defense system that counteracts damage from ROS, continuously generating and removing ROS to maintain a balance that ensures healthy cellular function and supports normal membrane function and the processing of cellular metabolites. This antioxidant defense system is broadly divided into two categories: enzyme-based defense mechanisms and non-enzymatic antioxidant defense mechanisms. Key antioxidant enzymes in the enzyme-based defense mechanism include SOD, catalase (CAT), and glutathione peroxidase (GPX). Because ROS have short lifespans and are generated in very localized areas, these antioxidant enzymes are distributed to complement each other in eliminating ROS. These enzymes are naturally produced by our bodies, and because supplying them externally is challenging, methods to maximize their endogenous production are being explored. SOD plays a crucial role by removing ROS generated when inhaled oxygen is used in various metabolic processes and is directly proportional to human lifespan. CAT helps break down H₂O₂, produced through the decomposition of O_2_^−^·, into water and oxygen using SOD. GPX, with the help of vitamin E, β-carotene, and flavonoids, removes HO·. Non-enzymatic antioxidant defense mechanisms include vitamin A, vitamin C, vitamin E, β-carotene, cysteine, coenzyme Q, uric acid, bilirubin, flavonoids, sulfhydryl groups, and thioether compounds. These substances react with, and neutralize the toxicity of, non-enzymatic ROS such as HO· and ^1^O₂. Unlike the body’s endogenous enzymes like SOD, CAT and GPX, these non-enzymatic defense substances can be supplied externally, making them a significant focus of modern anti-aging therapies [20,21,22,23,24,25]. There remain clear limitations in current treatment strategies for facial nerve injury. We hypothesize that reactive oxygen species (ROS) and free radicals (FRs) play critical roles in both nerve degeneration and regeneration, and speculate that a comprehensive synthesis of existing studies may help identify potential therapeutic pathways to support facial nerve recovery.

## 2. Research Methods

**Figure 1:**
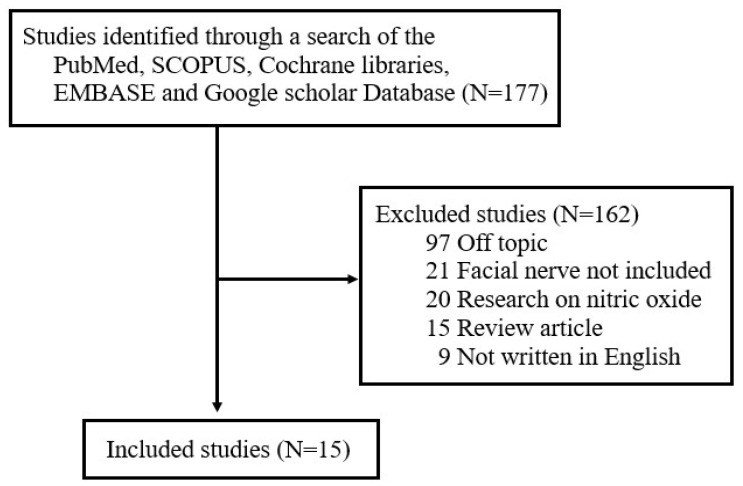
Review flow diagram.

Although various studies have examined the role of FRs and ROS in different diseases, there has not been a comprehensive review of the literature on the production and role of FRs and ROS after facial nerve injury. To address this unmet need, one author (J.L) searched for studies published between January 1997 and September 2024 in five electronic databases—Cochrane Libraries, EMBASE, Google Scholar, PubMed, and SCOPUS—using the search terms ‘free radicals’, ‘reactive oxygen species’, and ‘facial nerve’. The literature search focused on studies published in English and included (1) prospective or retrospective studies on NO in facial nerves and (2) studies involving either humans or animals. The following exclusion criteria were applied: (1) unpublished data, (2) review articles, (3) gray literature, (4) case reports, (5) duplicates, and (6) previously published literature review studies by the author on the expression of nitric oxide (NO) in facial nerves [16]. Of the original 157 studies returned by search terms, 15 were ultimately selected for review (Figure 1). All selected studies focused on analyzing changes in FR and ROS levels in the context of facial nerve injury. The papers were categorized based on whether FRs and ROS played a positive or negative role in the pathogenesis of facial nerve degeneration and regeneration.

## 3. Results

### 3.1. Facial Nerve Degeneration Through Increased ROS and FR After Facial Nerve Injury

Studies on the involvement of ROS and FRs in nerve degeneration after facial nerve injury have utilized various methods to induce facial nerve damage. These methods include physical techniques such as transection and compression, viral infections, and ischemic injury through vessel embolization. Because ROS are highly unstable and difficult to measure directly, oxidative stress is indirectly assessed by measuring markers of lipid peroxidation induced by ROS, such as malondialdehyde (MDA), as well as substances generated by ROS-induced ferroptosis. The modulation of oxidative stress using antioxidant scavengers, including alpha-lipoic acid (ALA), edaravone, N (ω)-nitro-L-arginine methyl ester (L-NAME), glutathione peroxidase 4 (GPX4), glutathione (GSH), methylprednisolone sodium succinate (MPSS), Si-based agents, SOD, and tirilazad mesylate [26,27,28], has also been studied.

#### 3.1.1. Peroxynitrite, NO, and NOS

In a study involving male Sprague Dawley rats subjected to left facial nerve transection [29], researchers found that NO and peroxynitrite, together with other NO donors, inhibited the polymerization activity of tubulin. Of these, peroxynitrite was the more potent inhibitor of tubulin polymerization. Treatment with the NO inhibitor L-NAME enhanced the polymerization activity of tubulin and the ability of tau to promote microtubule assembly. These findings suggest that NO and peroxynitrite inhibit the polymerization of facial nerve tubulin and disrupt the role of tau in microtubule assembly. This indicates that suppressing NO and peroxynitrite may facilitate axon regeneration and recovery following peripheral nerve injury [29]. Another study employing adult female Wistar rats investigated differences in the degree of damage and expression levels of FR-related enzymes depending on the site of facial nerve injury [30]. In this study, the expression of FR-related enzymes and the severity of cell loss was assessed after performing a distal axotomy at the stylomastoid foramen and a proximal axotomy at the brainstem surface. Neurons with distal lesions showed an increase in nitric oxide synthase (NOS) expression, together with a persistent decrease in the expression of calcineurin, an enzyme that activates NOS. There was also a transient increase in manganese-dependent SOD (Mn-SOD) expression, accompanied by mild neuronal loss. In contrast, proximal axotomy led to an increase in NOS expression, but a transient decrease in calcineurin and Mn-SOD expression at 4 weeks post-injury. Compared with distal axotomy, proximal axotomy resulted in earlier morphological changes in glial cells, including the hypertrophy of astrocytic processes and the transformation of ramified microglia into amoeboid cells. Moreover, degeneration progressed more rapidly following proximal axotomy. These results suggest that proximally axotomized facial motor neurons (FMNs) are more vulnerable to superoxide toxicity than distally axotomized neurons [30].

#### 3.1.2. Erastin-Induced Ferroptosis

Several studies have reported that ferroptosis plays a critical role in central nervous system diseases such as stroke, Parkinson’s disease, Alzheimer’s disease, and Huntington’s disease. These studies have shown that ferroptosis is closely associated with increased lipid peroxidation, decreased GSH levels, reduced GPX4 activity, and increased prostaglandin-endoperoxide synthase 2 (PTGS2) expression, all of which contribute to neuronal cell death.

In one study that examined the role of c-Jun in ROS-related ferroptotic responses to facial nerve injury [31], the major branches of the facial nerve were exposed by making a right postauricular incision after anesthesia. An adeno-associated virus (AAV) expressing c-Jun or small interfering RNA (siRNA) targeting c-Jun, or an empty vector (control), was then injected into the nerve tissue using a microinjector. After allowing 3 weeks for viral expression, a 50 s compression injury was applied to the facial nerve. Erastin, a substance known to increase intracellular ROS levels and induce ferroptosis, was administered after facial nerve injury. Erastin administration led to a reduction in GPX4, c-Jun protein, and GSH levels, while increasing levels of PTGS2, nuclear factor erythroid-2-related factor 2 (NRF2), and hemeoxygenase-1 (HO-1) proteins, as well as MDA, Fe^2+^ and ROS levels. The AAV-mediated overexpression of c-Jun exerted a protected effect against erastin-induced ferroptosis, whereas the siRNA-mediated downregulation of c-Jun had the opposite effect. These findings indicate that erastin-induced ferroptosis contributes to worsening neuronal damage in facial nerve injury through increased ROS levels, MDA accumulation, GSH depletion, and diminished c-Jun expression.

#### 3.1.3. Unscheduled DNA Synthesis and Mitochondrial DNA Synthesis

One study examined unscheduled DNA synthesis (UDS) and mitochondrial DNA (mtDNA) synthesis rates after right facial nerve transection [32]. This study, performed in adult male Wistar rats, detected a significant increase in UDS in FMNs 12 h after axotomy, and observed a notable increase in UDS and mtDNA synthesis in the regenerating facial nucleus 4 days after axotomy. Two weeks after axotomy, the mRNA expression of O6-alkylguanine-DNA-alkyltransferase (AGT), which repairs alkylated DNA by removing alkyl groups from the O6 position of guanine, was measured using Northern blot analysis. AGT mRNA expression was identical on both the axotomized and normal side, suggesting that DNA repair activity was not triggered by endogenously produced alkylating agents. The transient increase in UDS, enhanced mtDNA synthesis, and elevated protein synthesis rates observed in regenerating motoneurons indicate that FRs generated in the mitochondria of damaged neurons cause nonspecific DNA damage during the regeneration process.

Collectively, these studies demonstrate that ROS and FRs play a crucial role in nerve degeneration following facial nerve injury. Moreover, their involvement is linked to oxidative stress, nitrosative stress, and ferroptosis. These processes promote neuronal damage and reduce cell survival rates, highlighting the therapeutic potential of controlling ROS and FR levels as a strategy for promoting nerve injury recovery (Table 1 and Table 2).

### 3.2. Facial Nerve Regeneration by Antioxidants After Facial Nerve Injury

Several studies have investigated the effects of antioxidant administration following facial nerve injury, with the goal of preventing ROS- and FR-induced nerve degeneration while promoting recovery. The primary endogenous enzymatic scavengers that mitigate oxidative stress include SOD and glutathione (GSH). Other antioxidants, including edaravone, a potent scavenger of hydroxyl radicals; tirilazad mesylate (U-74006F), an inhibitor of lipid peroxidation; alpha-lipoic acid (ALA); and Si-based agents, have been employed to indirectly demonstrate the critical role of ROS and FR-induced oxidative stress in nerve injury processes.

#### 3.2.1. Superoxide Dismutase

Peripheral nerve injury induces genetic changes in the expression of various neuroactive substances within the cell soma that are known to play roles in adaptation to damage, neuronal survival, growth, and regeneration. In one study examining the effect of the systemic administration of SOD following the transient paralysis of the facial nerve induced by vascular embolization [33], ischemic facial paralysis was induced in male Sprague Dawley rats by injecting embolizing material into the right-side vessels supplying the temporal bone. Rats were divided into four groups—ischemia + saline, ischemia + SOD, axotomy + saline, and axotomy + SOD—and the severity of facial paralysis and degree of recovery was assessed, together with the expression of calcitonin gene-related peptide (CGRP), c-Jun, and growth-associated protein (GAP)-43 mRNA. After facial injury, the induction of CGRP, c-Jun, and GAP-43 mRNA was significantly greater in the axotomy group than in the embolization group (*p* < 0.01). SOD treatment accelerated recovery in the complete paralysis group compared to the control group, in association with a significant reduction in CGRP mRNA expression. These findings indicate that FRs play an essential role in peripheral nerve injury after ischemia and suggest that SOD may have a protective effect in nerve injury [34].

#### 3.2.2. SOD and GSH

Based on the previous identification of human herpesvirus 7 (HHV7) as a potential cause of Bell’s palsy, researchers conducted a study in 23-week-old female Wistar rats in which HHV7 infection was used to induce facial nerve palsy [35]. Following HHV7 inoculation, there was a significant increase in MDA and COX activity—both lipid peroxidation markers—together with an increase in the expression of COX4I2 (complex IV subunit 4 isoform 2), which promotes ROS production. Ferroptosis-related genes were also significantly upregulated in the experimental group compared with the control group (*p* < 0.01), whereas the antioxidant factors, SOD and GSH, were significantly decreased (*p* < 0.01). The increase in MDA and the decrease in SOD expression following facial nerve injury suggest that ROS-induced oxidative stress plays a crucial role in nerve degeneration.

#### 3.2.3. L-NAME

We investigated NOS/NADPH-diaphorase (NADPH-d) activity following facial nerve injury and the potential therapeutic benefit of L-NAME in a study on 28 Wistar albino rats subjected to left facial nerve avulsion [36]. In unoperated facial motoneurons, NADPH-d staining was faint and NOS immunoreactivity was absent. However, after facial nerve avulsion, NADPH d reactivity increased progressively, with NOS-positive neurons emerging by day 5. Both markers reached their peak staining intensity on day 20, after which the number of NOS-positive neurons began to decline in parallel with a decrease in surviving facial motoneurons. By day 50 of the experiment, all surviving neurons were NADPH-d positive, but only half were NOS positive. Notably, daily administration of L-NAME reduced neuronal loss by approximately 17% compared with the control group, suggesting that NO has neuro-destructive properties. These findings indicate that NADPH-d/NOS plays a crucial role in neuronal injury and that NOS inhibition may help to mitigate neuronal loss.

#### 3.2.4. Methylprednisolone Sodium Succinate

The effect of high-dose (160 mg/kg) methylprednisolone sodium succinate (MPSS) on endogenous NO formation in the brainstem and its impact on the survival of facial motoneurons following facial nerve transection were evaluated in 83 Hartley albino guinea pigs [37]. The results showed that NO formation was significantly increased in the facial nerve transection group compared to the control group, and that NO formation was linearly correlated with nNOS-positive neurons (*r* = 0.934, *p* = 0.0007). Moreover, NO formation was negatively correlated with neuronal survival, indicating that increased NO formation is associated with neuronal loss. Three to four weeks post-transection, the survival rate of motor neurons was significantly higher in the MPSS-treated group than in the control group. These results suggest that MPSS delays NO formation and improves motor neuron survival, demonstrating a strong correlation between neuronal damage and NO formation.

#### 3.2.5. Edaravone

The role of NO in HSV-1-induced facial nerve paralysis and the potential of edaravone to prevent facial palsy were examined in a study conducted using 62 female Balb/cAjcl mice [38]. Seven days after HSV-1 inoculation, NO levels and the incidence of transient facial palsy were significantly higher in the inoculated side than in the control side (*p* < 0.001). NO levels decreased as facial palsy recovery progressed; in cases where transient facial palsy did not develop, no increase in NO was observed. Notably, the intraperitoneal administration of edaravone, a potent FR scavenger, significantly reduced the incidence of facial palsy. These findings suggest that NO plays a critical role in the progression of HSV-1-induced facial palsy and that edaravone may aid in its recovery. Another study investigated the role of edaravone in ischemic facial palsy induced by petrosal artery interruption [39]. Using immunofluorescence analysis, this study, employing 67 guinea pigs, revealed increased ROS expression in facial nerve samples from animals that did not receive edaravone treatment. In contrast, ROS expression was significantly reduced in samples from animals who were treated with intraperitoneal edaravone (*p* < 0.001). The edaravone-treated group exhibited a lower incidence of facial palsy, reduced tissue damage, and the attenuation of degenerative changes in the facial nerve. Two weeks after injury, the myelin sheath appeared irregular, but the intrinsic axonal structure remained intact. By the 4-week follow-up point, myelin had recovered well. These findings indicate that edaravone has the potential to prevent the onset and progression of ischemia-induced facial palsy and that ROS play a crucial role in facial palsy development.

#### 3.2.6. Tirilazad Mesylate

The effects of tirilazad mesylate (U-74006F), a lipid peroxidation inhibitor, on motoneuronal degeneration following right facial nerve transection was investigated in a study using male Sprague Dawley rats [40]. Treatment with U-74006F increased the percentage of motor neuron survival and reduced retrograde degeneration relative to controls. These results suggest that the inhibition of lipid peroxidation can protect motor neurons from post-axotomy degeneration, highlighting the role of oxygen radical-induced membrane peroxidation in apoptotic neuronal death.

#### 3.2.7. Alpha-Lipoic Acid

Changes in the expression of NADPH oxidase 2 (NOX2) and the degree of functional recovery following different types of facial nerve injury, as well as the therapeutic value of ALA in facial nerve regeneration, were investigated in a study conducted on 40 mature male Sprague Dawley rats [41]. The animals were randomly divided into four groups: (A) crushing injury only, (B) crushing injury with ALA, (C) axotomy only, and (D) axotomy with ALA. Regardless of injury type, NOX2 expression ratios were significantly higher in ALA-injected groups than in non-injected groups (*p* < 0.001). Behavioral tests further showed higher vibrissae movement scores and blink reflex scores in the crushing injury group than in the axotomy group (*p* < 0.001). ALA administration significantly improved behavioral test scores in the crushing injury group compared to the non-treated group (*p* = 0.031). These findings suggest that ALA may aid in peripheral facial nerve injury recovery.

#### 3.2.8. Si-Based Agent and MeCbl Combination Therapy

Silicon (Si)-based agents are substances that react with water to continuously generate hydrogen, which exhibits antioxidant properties. Methylcobalamin (MeCbl) is an active form of vitamin B12 known to promote nerve regeneration. To investigate the effects of Si-based agents and methylcobalamin (MeCbl) combination therapy on facial nerve crush injuries, researchers performed a study using C57BL/6J mice with left facial nerve compression injuries [42]. The mice were divided into four groups: (1) untreated group fed a control diet (Con group), (2) untreated group fed a diet containing a Si-based agent (Si group), (3) MeCbl-treated group fed a control diet (MeCbl group), and (4) MeCbl-treated group fed a diet containing a Si-based agent (Si/MeCbl group). The group administered the Si-based agent exhibited faster recovery of facial nerve function, enhanced myelin sheath formation, and reduced oxidative stress compared with the untreated group. ROS levels were significantly higher in the control group, but were not substantially increased in the Si group. Notably, ROS elevation was most significantly suppressed in the Si/MeCbl group. These findings suggest that Si-based agents facilitate myelin sheath formation and inhibit oxidative stress, thereby aiding facial nerve regeneration. This study further highlights the crucial role of ROS in facial nerve degeneration.

These findings collectively confirm the ability of various antioxidants to effectively prevent the progression of nerve damage caused by ROS and FR while promoting recovery, underscoring the importance of oxidative stress as a key pathological mechanism in nerve injury. Such research results suggest the potential future use of antioxidants as a therapeutic strategy for facial nerve injury and degeneration (Table 3).

### 3.3. Age-Dependent Role of ROS and FR in Facial Nerve Injury

In an earlier study that investigated the effects of facial nerve transection at different postnatal ages [43], researchers assessed NADPH-d positivity as a proxy for NOS activity. Prior to facial nerve transection, NADPH-d positivity was undetectable in 31 Wistar male rats of different postnatal ages (birth to postnatal month 3). However, after performing a facial nerve transection on the left side, significant postnatal age-dependent differences in NOS induction were noted in facial motoneurons. When the axotomy was performed immediately after birth, no NADPH-d positivity was observed for the first 1–2 days, but moderate NADPH d staining was detected by day 4. In cases where the axotomy was performed during the first or second postnatal week, no NADPH-d positivity was seen until day 2; by day 4, distinct and strong NADPH-d staining was observed. When the axotomy was conducted on postnatal day 14, we found that only a small number of facial motoneurons exhibited strong NADPH-d positivity by day 4. In this case, the facial nucleus showed moderate overall staining intensity. This that weaker than that observed following the axotomy performed during the first postnatal week. When axotomy was performed at postnatal week 5, NADPH-d induction in the injured facial motoneurons was very weak or nearly absent on day 4 after the injury, a finding unlike that observed in the first two postnatal weeks. By day 7 after injury, distinct NADPH-d positivity began to appear in the injured facial nucleus, and its staining intensity gradually increased over time. By day 20 post-injury, very strong NADPH-d staining was observed in the cell bodies of injured facial motoneurons. By this time, approximately one-third of all motoneurons had been lost in the injured compared with the uninjured side. In cases where the axotomy was performed at 2 or 3 months of age, NADPH-d positivity appeared strongly in the injured motoneurons by day 7, with particularly intense staining in the cell bodies and proximal dendrites of the facial motoneurons on the same side as the injury. These results suggest the existence of age-dependent differences in NOS expression after axotomy and imply that NOS induction may not be an essential factor in motoneuronal cell death following injury. They further indicate that NOS induction and the death of immature motoneurons at a distance from their target may be unrelated phenomena (Table 4 and Table 5).

### 3.4. Molecular Mechanisms of ROS and Free Radicals in Facial Nerve Injury

Reactive oxygen species (ROS) and free radicals (FRs) significantly impact various key intracellular signaling pathways in neural tissue following facial nerve injury. Notably, pathways such as MAPK (Mitogen-Activated Protein Kinase), NF-κB (nuclear factor kappa-light-chain-enhancer of activated B cells), PI3K/Akt, and NRF2/HO-1 are activated or modulated. These signaling pathways intricately regulate physiological processes such as cell death, inflammatory responses, and cell survival post-nerve injury [44,45,46]. Furthermore, ROS and FRs closely interact with various growth factors and cytokines, which play crucial roles in nerve regeneration. Specifically, increased ROS levels promote the secretion of pro-inflammatory cytokines such as interleukin-1 beta (IL-1β) and tumor necrosis factor-alpha (TNF-α), exacerbating inflammation and accelerating neuronal degeneration. Conversely, appropriate levels of ROS can activate the signaling pathways favorable for nerve regeneration by promoting the activity of growth factors like NGF (Nerve Growth Factor), BDNF (Brain-Derived Neurotrophic Factor), and GDNF (Glial Cell line-Derived Neurotrophic Factor) [47,48,49]. Lipid peroxidation induced by ROS is one of the critical molecular mechanisms leading to nerve damage and is triggered by the oxidation of polyunsaturated fatty acids present in neuronal cell membranes. This oxidative process forms toxic aldehydes such as malondialdehyde (MDA) and 4-hydroxynonenal (4-HNE), which damage the structure and function of cell membranes. Additionally, ferroptosis induced by ROS occurs due to glutathione depletion and the decreased activity of GPX4 (Glutathione Peroxidase 4), generating iron-dependent lipid radicals that ultimately promote neuronal cell death. Mitochondrial dysfunction represents another key pathway of nerve damage caused by ROS and FRs. ROS directly damage mitochondrial DNA (mtDNA), proteins, and lipids, impairing the function of the electron transport chain and reducing ATP production. This mitochondrial damage creates a vicious cycle that further enhances ROS production, exacerbating nerve damage. ROS also significantly influence intracellular gene expression by regulating key transcription factors such as NF-κB, AP-1 (Activator Protein-1), NRF2 (nuclear factor erythroid-2-related factor 2), and HIF-1α (Hypoxia-inducible factor 1-alpha), thereby altering the expression of genes related to oxidative stress response, inflammation, cell death, and survival [50,51].

The neural response to ROS and FRs varies with the location of injury and age, which can be explained at the molecular level. Different outcomes are observed depending on the site of injury due to regional differences in antioxidant enzyme (Mn-SOD, CAT) distribution, calcium homeostasis, and the inherent vulnerability of each neural cell. Additionally, with aging, the activity of antioxidant enzymes decreases, DNA repair capacity diminishes, and the expression of ROS-sensitive signaling proteins changes, increasing susceptibility to oxidative stress. The extent to which neurons respond to the toxicity of ROS and FRs is determined by several factors, including the expression level of antioxidant enzymes, mitochondrial integrity, metabolic demands, calcium buffering capacity, and the intrinsic vulnerability of different neuronal cell types. Glial cells also actively respond to ROS, significantly impacting neuronal health. Microglia become activated in the presence of ROS, releasing inflammatory cytokines (TNF-α, IL-1β), chemokines, and neurotoxic mediators such as nitric oxide (NO), further aggravating nerve damage. Conversely, astrocytes upregulate antioxidant enzymes like SOD and GPX4, partially mitigating nerve damage caused by oxidative stress [52,53,54,55].

## 4. Limitations

This study holds academic significance as the first comprehensive literature review on the pathophysiological roles of reactive oxygen species (ROS) and free radicals (FRs) following facial nerve injury (FNI). However, it has several limitations.

Firstly, the studies included in this review are primarily based on experimental research using animal models, with clinical studies involving humans being very limited. Further research is needed to determine if the expression patterns of ROS and FRs and the therapeutic responses to antioxidants observed in animal models are consistent in humans. Physiological characteristics, such as oxidative stress response, nerve regeneration rate, and inflammatory response, can significantly differ among species. Therefore, caution is required when applying these findings directly to humans. Moreover, in clinical research, ethical constraints prevent the intentional induction of nerve injuries as occurs in animal experiments, and the use of invasive approaches is restricted. Additionally, the difficulty in repeating experimental designs or conducting repeated experiments under various conditions can limit the reproducibility and generalizability of the research. Thus, robust scientific evidence is necessary to apply animal experiment results to clinical practice with actual patients. Secondly, there is considerable methodological heterogeneity among the reviewed studies. Variations in experimental models for inducing facial nerve injury (e.g., transection, compression, ischemia, viral infection), types of animals used (e.g., Sprague Dawley rats, guinea pigs), injury locations (proximal vs. distal), and types and methods of antioxidant administration present challenges when comparing and interpreting the results comprehensively. Furthermore, many included studies have limited sample sizes, which can undermine the statistical reliability and reproducibility of the findings. Small sample sizes may lead to random biases and limit the generalization of the results to a broader context. Thirdly, the heterogeneity in the methods of measuring ROS and FRs across studies posed difficulties in comparison. Standardization in the collection, storage, and analysis of biological samples is needed. Using the same analytical kits, reagents, and reference standards to measure lipid peroxidation markers and antioxidant enzyme activities could facilitate the interpretation of results across studies. Additionally, it is crucial to employ and standardize advanced direct detection technologies, such as Electron Paramagnetic Resonance (EPR) spectroscopy, fluorescence-based ROS detection methods (e.g., DCF-DA, MitoSOX), and genetically encoded ROS sensors (e.g., HyPer), to accurately measure temporal and spatial concentrations. Fourthly, most studies only focus on short-term results over days to weeks, making it difficult to conclusively determine the long-term effects of ROS modulation or antioxidant therapy on facial nerve regeneration and functional recovery. Future studies should include long-term follow-up observations to clearly assess the sustained effects of treatment and long-term nerve regeneration outcomes. Fifthly, biological variables such as age and sex can influence ROS and FR reactions, but most studies have not sufficiently controlled for these variables or have only addressed them to a limited extent. Although there are reports indicating that NADPH-diaphorase and NOS expression varies with age, comprehensive analyses of these biological variables are lacking. Additional studies incorporating these variables are needed in the future.

These limitations must be considered when interpreting the results of this paper and applying them clinically. Future research should aim to establish more standardized experimental protocols, develop direct ROS measurement methodologies, and conduct long-term and large-scale clinical studies to address these issues.

**Table 5:** Age-dependent role of ROS and FR in facial nerve injury.

Author/ Year/ Reference	Study Design	Species and/or Sample	Nerve/Injury Method	Detection Method	Target Substance(s) Associated with Free Radicals	Results/Conclusions
Mariotti, R. et al., 1997 [43]	Animal study	31 Wistar male rats	Unilateral facial nerve transection (left side)	NADPH-d histochemistry, TUNEL assay	NOS, NADPH-d	Facial motoneurons in the non-operated side showed no evidence of NADPH-d positivity at any examined age, whereas most experiments showed positive NADPH-d staining in axotomized facial motoneurons. In cases where the facial nerve was cut on postnatal day 0, NADPH-d staining was undetectable in facial nuclei on either side after 1 or 2 days. For facial nerves transected 1 week postnatally, NADPH-d induction was absent in ipsilateral facial nucleus after 2 days, but was marked in injured facial motoneurons 4 days after the axotomy. NADPH-d activity was observed in facial motoneurons following axotomy, beginning from the end of the first postnatal week and continuing through adulthood, a period during which neuronal loss was less pronounced compared to that in newborns. The time course of NADPH-d induction in facial motoneurons following postnatal axotomy exhibits remarkable age dependence. By contrast, 1 week was sufficient to observe an intense NADPH-d induction in facial motoneurons axotomized in the adult. These data support the view that NOS induction in motoneurons requires a certain amount of time after nerve injury and highlight the age-dependent nature of the temporal gradient.

Abbreviations: NOS, nitric oxide synthase; NADPH-d, reduced nicotinamide adenine dinucleotide phosphate dehydrogenase; TUNEL, terminal deoxynucleotidyl transferase-mediated dUTP nick-end labeling.

## 5. Conclusions

To investigate the production and role of ROS in facial nerve injury, we conducted a review of 15 studies sourced from SCOPUS, PubMed, the Cochrane Library, EMBASE, and Google Scholar. Facial nerve injury triggers a variety of molecular and cellular responses, with oxidative stress induced by ROS playing a critical role in both nerve damage and the recovery process. Excessive ROS-induced oxidative stress, observed in various facial nerve injury models, leads to mitochondrial dysfunction, lipid peroxidation, and, subsequently, ferroptosis, and apoptosis.

**Figure 2:**
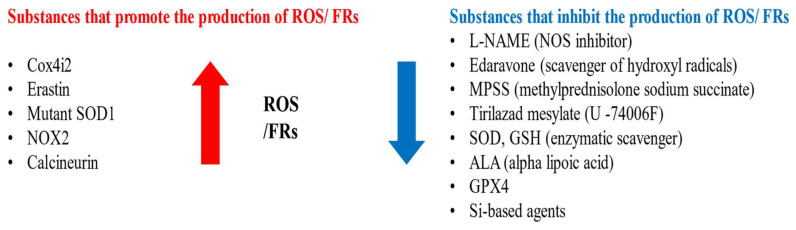
Substances that promote or inhibit the production of FRs and ROS.

**Figure 3:**
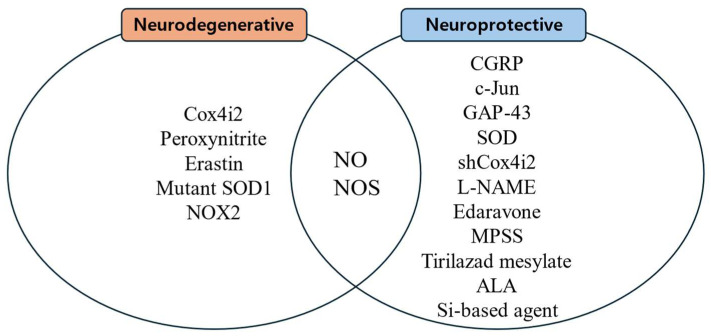
Substances related to nerve regeneration and nerve degeneration.

**Figure 4:**
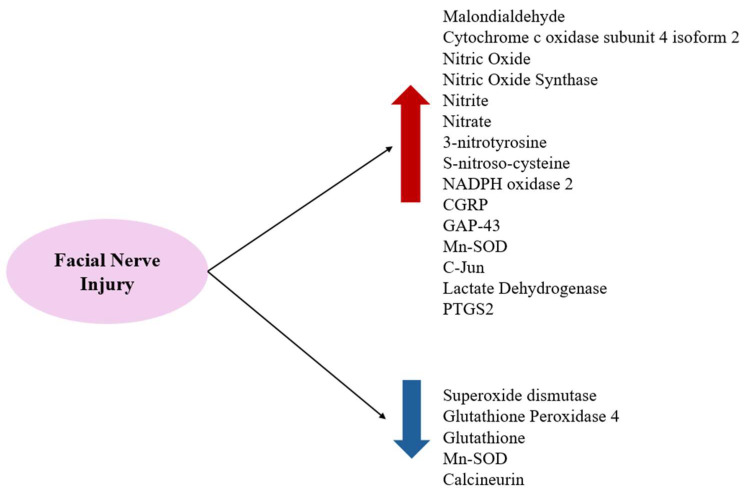
FRs and ROS increase after facial nerve injuries and antioxidant-related substances decrease.

Substances that increase ROS levels in experimental settings include COX4I2, erastin, and NOX2, all of which have been shown to exacerbate facial nerve injury. Conversely, compounds that regulate or mitigate ROS levels, such as ALA, edaravone, L-NAME, GPX4, GSH, MPSS, silicon-based agents, SOD, and tirilazad mesylate, are found to prevent the worsening of nerve injury and promote nerve regeneration. Additionally, ROS expression varies depending on the location of the nerve injury, with proximal lesions resulting in more extensive neuronal damage and greater susceptibility to oxidative stress toxicity than distal lesions (Figure 2, Figure 3 and Figure 4).

In conclusion, the effects of ROS and FRs in facial nerve injury are influenced by factors such as the injury location, the age of the experimental animals, detection methods, and the use of indirect measures of enzymes and antioxidants. ROS play an essential role in the pathophysiology of facial nerve injury, and maintaining balanced ROS levels is critical for nerve recovery. Therapeutic approaches involving antioxidant supplementation, the regulation of scavenger enzymes, and the inhibition of ROS pathways are expected to be beneficial for the treatment of facial nerve injuries. Future studies are needed to further elucidate the precise mechanisms of ROS and FRs in facial nerve injury and explore strategies for their regulation. In addition, it should be noted that all the studies in this review were conducted using animal models, and further research involving human cohorts is necessary to validate these findings and translate them into clinical practice.

## Figures and Tables

**Table 1:** The production of substances related to ROS and FRs in the context of nerve degeneration and regeneration after facial nerve injury.

Type of Injury	Increased	Decreased
Viral infection	c-Jun COX4I2 LDH MDA NO NO_2_^−^ (nitrite), NO_3_^−^ (nitrate) NOS PTGS2	GSH GPX4 SOD
Axotomy	c-Jun CGRP GAP-43 Mn-SOD (after distal axotomy) NO NOS NOX2 3-NT, SNO-Cys	Calcineurin Mn-SOD (after proximal axotomy)

Abbreviations: CGRP, calcitonin gene-related peptide; COX4I2, cytochrome c oxidase subunit 4 isoform 2; GAP-43, growth-associated protein 43; GSH, glutathione; GPX4, glutathione peroxidase 4; NO, nitric oxide; NOS, nitric oxidase synthase; NOX2, NADPH oxidase 2; MDA, malondialdehyde; Mn-SOD, manganese superoxide dismutase; PTGS2, prostaglandin-endoperoxidase synthase 2; LDH, lactate dehydrogenase.

**Table 2:** Facial nerve degeneration by increased ROS and FR after facial nerve injury.

Author/ Year/ Reference	Study Design	Species and/or Sample	Nerve/Injury Method	Detection Method	Target Substance(s) Associated with Free Radicals	Results/Conclusions
Yeh, T.Y. et al., 2021 [29]	Animal study	Male Sprague Dawley rats	Left facial nerve transection	L-NAME treatment, immunoprecipitation, microtubule assembly assay, immunohistochemistry	NO, peroxynitrite, 3-NT, SNO-Cys	3-NT and SNO-Cys (nitrosative stress biomarkers) were highly upregulated in proximal and distal stumps of reconnected facial nerves 3 days and 1 week after neurorrhaphy. 3-NT expression was substantially reduced at 2 weeks, whereas that of SNO-Cys was maintained. The inhibition of NO production with L-NAME prevented the upregulation of SNO-Cys and enhanced the polymerization activity of tubulins and the ability of tau to promote microtubule assembly. NO or peroxynitrite reduces the polymerization of normal rat facial nerve tubulins and the ability of tau to promote microtubule assembly. Preventing NO- or peroxynitrite-induced alterations in tubulin or tau may be clinically useful for facilitating microtubule assembly and the consequent recovery of axon regeneration.
Liu, P.H. et al., 2006 [30]	Animal study	Adult female Wistar rats	Distal and proximal facial axotomy	Light microscopy, electron microscopy, immunohistochemistry, TUNEL assay, cresyl-violet staining	nNOS, calcineurin, Mn-SOD	Distal lesions resulted in the upregulation of NOS expression in neurons and the persistent downregulation of neuronal expression of the NOS-activating enzyme calcineurin. They also led to the transient downregulation of neuronal expression of Mn-SOD and modest neuronal loss. Proximal axotomy led to the upregulation of NOS but transient downregulation in the expression of calcineurin and Mn-SOD at 4 weeks after injury. nNOS expression in proximally axotomized FMNs was more likely to produce NO, beginning 1 week after lesioning, than nNOS in distantly axotomized FMNs if other intracellular factors that affected nNOS function were not greatly altered in the two cases. The mitochondrial Mn-SOD expression pattern in proximally axotomized FMNs further suggests that these mitochondria are more likely to face serious superoxide toxicity than those in distally axotomized cells.
Gao, D. et al., 2022 [31]	Animal study	Sprague Dawley rats	Right facial nerve injury with AAV-mediated expression of c-Jun or c-Jun siRNA	Immunofluorescence, cell viability, PI staining, ROS, ELISA, lentivirus transfection, Western blotting, RT-PCR	c-Jun, GPX4, GSH, PTSG2, MDA, Fe^2+^, ROS	The ferroptosis activator erastin decreased GPX4 and c-Jun protein expression and GSH content, while increasing PTSG2, NRF2, and HO-1 protein, and MDA, Fe^2+^, and ROS content. These effects were inhibited by c-Jun overexpression but were reversed by siRNA-mediated c-Jun knockdown. c-Jun can inhibit ferroptosis in the facial nerve and promote facial nerve recovery and S-100 expression, allowing the nerve to form a more morphologically normal myelin sheath.
Korr, H. et al., 1997 [32]	Animal study	Adult male Wistar rats	Right facial nerve transection	Light microscopic autoradiography, Northern blotting, electron microscopic autoradiography	FRs	In addition to the increased rate of mtDNA synthesis in facial motoneurons 12 h after axotomy, UDS and mtDNA synthesis significantly increased in the regenerating facial nucleus 4 days after axotomy. FRs produced by mitochondria in injured nerve cells can cause nonspecific DNA damage, followed by immediate repair.

Abbreviations: AAV, adeno-associated virus; ELISA, enzyme-linked immunosorbent assay; FMN, facial motor neuron; FR, free radical; GSH, glutathione; GPX4, glutathione peroxidase 4; HO-1, heme oxygenase-1; L-NAME, N-nitro-L-arginine methyl ester; MDA, malondialdehyde; Mn-SOD, manganese-dependent superoxide dismutase; NO, nitric oxide; NOS, nitric oxide synthase; NRF2, nuclear factor erythroid-2-related factor 2; PI, propidium iodide; ROS, reactive oxygen species; RT-PCR, reverse transcription polymerase chain reaction; siRNA, small interfering RNA; SOD, superoxide dismutase; SNO-Cys, anti-S-nitroso-cysteine; TUNEL, terminal deoxynucleotidyl transferase-mediated dUTP nick-end labeling; UDS, unscheduled DNA synthesis; 3-NT, 3-nitrotyrosine.

**Table 3:** Facial nerve regeneration by antioxidants after facial nerve injury.

Author/ Year/ Reference	Study Design	Species and/or Sample	Nerve/Injury Method	Detection Method	Target Substance(s) Associated with Free Radicals	Results/Conclusions
Mohri, D. et al., 2001 [33]	Animal study	Male Sprague Dawley rats	The induction of the transient paralysis of the facial nerve by the embolization of vessels supplying the temporal bone	Confocal laser-scanning microscopy, in situ hybridization, histochemistry	SOD	This study examining the effect of systemic administration of SOD on facial paralysis and mRNA expression in facial nerve nuclei following vascular embolization showed that CGRP, c-Jun, and GAP-43 mRNA exhibited distinct patterns of induction and a time course of increase after ischemic nerve injury (*p* < 0.05). SOD treatment clearly alleviated behavioral impairments and decreased CGRP mRNA expression on day 3 after injury (*p* < 0.01). Ischemic facial nerve injury can be attenuated by pre-treatment with SOD, providing indirect evidence that FRs may be partially responsible for the development of peripheral nerve injury after ischemia.
Mariotti, R. et al., 2002 [34]	Animal study	Mice carrying a mutated SOD1 gene and wild-type mice (B6SJL)	Axotomy of FALS facial motoneurons	NADPH-d histochemistry, immunohistochemistry, avidin-biotin peroxidase protocol, cresyl violet staining	SOD1, NOS	Axotomy elicited high NOS expression in facial motoneurons of wild-type mice after 2–3 weeks, whereas induction was very weak or absent in *Sod1*-mutant transgenic mice. At 1 month post-axotomy, the loss of facial motoneurons was significantly higher in mutant mice than in wild-type littermates. SOD1 mutation interferes with the oxidative cascade elicited by axonal injury in cranial motoneurons. Moreover, the adverse gain of function of mutant SOD1 enhances the vulnerability of motoneurons to peripheral stressful conditions.
Chang, B. et al., 2021 [35]	Animal study	23-week-old female Wistar rats	Induction of facial nerve injury by HHV7 inoculation	Cell culture and HHV7 infection, immunofluorescence staining, qRT-PCR, Western blotting, lipid peroxidation assay, flow cytometry, Phen green staining, TUNEL assay	SOD, MDA, GSH, COX4I2	HHV7-induced facial nerve injury increased levels of MDA and decreased levels of SOD and GSH, reflecting lipid peroxidation and weakening of antioxidant capacity. Transfection of shCox4i2 into HHV7-treated SCs relieved oxidative stress and decreased Cox4i2 expression. The same trend was observed for ROS concentrations (*p* < 0.01). The increased expression of Cox4i2 in HHV7-infected SCs promoted the production of ROS, whereas the knockdown of Cox4i2 expression in these cells caused a relative decrease in ROS levels.
Ruan, R.S. et al., 1995 [36]	Animal study	28 male Wistar albino rats	Left facial nerve avulsion	Immunocytochemistry, NADPH-d histochemistry	NOS, NADPH-d	In unoperated FMNs, NADPH-d staining in neurons was weak and NOS immunoreactivity was absent. After nerve injury, NADPH-d reactivity increased as early as 2 days, and NOS-positive neurons emerged by day 5. The intensity and number of both markers increased progressively, peaking at 20 days post-injury, after which NOS expression declined. By 30 days, >60% of neurons were lost in the operated FMNs, but neuronal loss was reduced to 43% by the daily administration of L-NAME. NADPH-d and NOS play roles in the neuronal response to injury, and NOS inhibition may protect against neuronal loss.
Chen, Y.S. et al., 2007 [37]	Animal study	83 Hartley albino guinea pigs	Right facial nerve transection	Immunohistochemistry, cresyl violet staining, NO/ozone chemiluminescence	nNOS, NO	Facial nerve transection induced a significant increase in NO formation in the brainstem by 1 week in both MPSS- and saline-treated groups that lasted to the end of the study (4 weeks). MPSS administration appeared to delay the increase in NOS expression and NO formation during the first 1–2 weeks after facial nerve transection compared with saline-treated controls (*p* < 0.001). The survival rate of FMNs was significantly higher in the MPSS-treated group than in the saline-treated group at 3 and 4 weeks after facial nerve transection (*p* < 0.05). The administration of a large dose of MPSS produces a delayed increase in NO formation in the brainstem that may attenuate the loss of FMNs.
Hato, N. et al., 2013 [38]	Animal study	62 female Balb/cAjcl mice	Facial paralysis induced by HSV-1 inoculation	Behavioral test, in vivo microdialysis, high-performance liquid chromatography	NO, iNOS, edaravone	In cases where HSV-1 induced facial palsy, which usually occurred 7 days after inoculation, NO levels were significantly higher on the inoculated side than on the control side (*p* < 0.001). High NO levels in the inoculated side decreased following recovery from palsy. No increase in NO levels was observed in animals without transient facial palsy. The administration of edaravone significantly decreased the incidence of facial palsy. NO produced by iNOS in the facial nerve plays an important role in the onset of facial palsy caused by HSV-1 infection, considered a causative virus of Bell’s palsy. This study elucidated the role of NO in HSV-1-related facial nerve paralysis in mice and evaluated the role of edaravone, an FR scavenger, in preventing paralysis
Takeda, T. et al., 2008 [39]	Animal study	Guinea pigs	Ischemic facial palsy induced by the interruption of the petrosal artery	Morphological study, light microscopy, electron microscopy, fluorescence microscopy	ROS, hydroxyl radicals	Edaravone (MCI-186), an extremely potent scavenger of hydroxyl radicals, inhibits not only hydroxyl radicals but also iron-induced peroxidative injuries. Edaravone injection produced signs of facial movement recovery beginning 15 to 19 days after the intervention. It also reduced tissue damage: nerve fibers were relatively intensely stained with Luxol fast blue and, significantly, cell bodies were observed in the geniculate ganglion. The excess generation of ROS likely participates in the development and severity of facial palsy, and the reduction of ROS could attenuate severe nerve damage.
Hall, E.D. et al., 1996 [40]	Animal study	14-day-old male Sprague Dawley rats	Facial nerve transection	Choline acetyltransferase immunocytochemistry, cresyl violet histochemistry	Lipid peroxidation inhibitor tirilazad mesylate (U-74006F)	Following facial nerve axotomy, survival rates of motor neurons in regions A, B, and C of the ipsilateral facial nucleus were 56.2%, 50.6%, and 57.4%, respectively, of those in the non-axotomized contralateral region (*p* < 0.001). Treatment with U-74006F significantly enhanced motor neuron survival, increasing survival rates in region B and C to 72.8% and 66.7%, respectively, at a dose of 10 mg/kg, and to 64.2% and 67.9% at a dose of 30 mg/kg. When limited to the first 5 days after axotomy, an oral 30 mg/kg dose of U-74006F also significantly blunted retrograde degeneration measured 21 days post-axotomy. The protection of axotomized FMNs by a lipid peroxidation inhibitor strongly suggests that neuronal apoptosis ultimately involves an oxygen radical-induced membrane peroxidative mechanism.
Yoo, M.C. et al., 2022 [41]	Animal study	40 mature Sprague Dawley rats	Left facial nerve crushing injury, axotomy	Behavioral tests, immunohistochemistry	NOX2, ALA	Relative NOX2 expression was higher in the axotomy group than in the crushing group (*p* < 0.001). Relative NOX expression trended higher in both injury groups that received an injection of ALA, but reached statistical significance only in the axotomy group (*p* < 0.001). Behavioral tests conducted 4 days after the crushing injury showed better results in the group injected with ALA than in the group without injection of ALA (*p* = 0.031). Injection with ALA promoted nerve regeneration in a rat model of crushing nerve injury. Moreover, NOX2 expression was significantly higher following facial nerve injury, particularly for a cutting injury.
Koyama, Y. et al., 2022 [42]	Animal study	C57BL/6J mice	Left facial nerve compression injury	Behavioral test, electroneuronography, oxidative stress measurement, immunofluorescence staining	Si-based agent, ROS	Combined treatment with a Si-based agent and MeCbl improved clinical scores and neuroregeneration and reduced oxidative stress compared with individual administration. The Si-based agent rapidly rescued the loss of normal facial expressions by promoting myelin sheath formation and alleviating oxidative stress. Combined Si-based agent/MeCbl therapy could be a clinically viable new treatment for facial paralysis.

Abbreviations: ALA, alpha-lipoid acid; CGRP, calcitonin gene-related peptide; FMN, facial motor neuron; FALS, familial amyotrophic lateral sclerosis; GAP-43, growth-associated protein 43; GSH, glutathione; HHV7, human herpes virus 7; HSV-1, herpes simplex virus 1; iNOS, inducible nitric oxide synthase; L-NAME, N-nitro-L-arginine methyl ester; MDA, malondialdehyde; MPSS, methylprednisolone sodium succinate; NADPH-d, reduced nicotinamide adenine dinucleotide phosphate dehydrogenase; NO, nitric oxide; NOS, nitric oxide synthase; NOX2, NADPH oxidase 2; ROS, reactive oxygen species; RT-PCR, reverse transcription polymerase chain reaction; SCs, Schwann cells; SOD, superoxide dismutase.

**Table 4:** Comparative summary of antioxidants: efficacy, potential advantages, and disadvantages.

Antioxidant	Efficacy	Potential Advantages	Potential Disadvantages
Alpha-lipoic acid (ALA)	Facilitates functional recovery in nerve injury models; inhibits NOX2 expression	Highly membrane-permeable; provides broad protection against multiple ROS/FR species	Mild adverse effects (e.g., gastrointestinal disturbances) at high doses
Superoxide dismutase (SOD)	Promotes recovery from ischemic injury; reduces pro-inflammatory gene expression	Highly effective in directly scavenging ROS, protective against nerve damage	Clinical application limited by administration route and short half-life
Glutathione (GSH)	Provides cytoprotection after nerve injury, inhibits ferroptosis	Potent antioxidant effect that promotes cell survival	Rapidly depleted in vivo; continuous replenishment strategies required
Edaravone	Prevents and mitigates ischemic and HSV-1-induced nerve palsy	Effectively scavenges various ROS, including hydroxyl radicals; rapid symptom alleviation	Possible side effects including hypersensitivity; cost and limited accessibility
Tirilazad mesylate (U-74006F)	Enhances motor neuron survival post-axotomy; inhibits lipid peroxidation	Specialized in protecting cellular membranes by effectively suppressing lipid peroxidation	Limited clinical studies; selective action limited to specific types of FRs
Methylprednisolone sodium succinate (MPSS)	Delays NO formation after nerve injury; improves motor neuron survival	Strong anti-inflammatory and antioxidant effects; effective in early-phase treatment of nerve injury	Risk of severe adverse effects such as infection, gastrointestinal bleeding with high-dose or long-term use
L-NAME (NOS inhibitor)	Reduces neuronal damage and neuronal loss through inhibition of NO synthesis	Potent neuroprotective effect when administered shortly after injury	Risk of interfering with essential physiological functions mediated by NO (e.g., vasodilation, neurotransmission)
Si-based agent and methylcobalamin (MeCbl)	Promotes nerve regeneration and reduces ROS levels after nerve compression injury	Dual beneficial effects through alleviating oxidative stress and enhancing myelin sheath regeneration	Insufficient clinical data; uncertainty regarding long-term efficacy with prolonged administration

## Data Availability

Not applicable.
